# National health financing policy in Eritrea: a survey of preliminary considerations

**DOI:** 10.1186/1472-698X-12-16

**Published:** 2012-08-28

**Authors:** Joses Muthuri Kirigia, Eyob Zere, James Akazili

**Affiliations:** 1 World Health Organization, Regional Office for Africa, Brazzaville, Congo; 2 UNFPA, Dhaka, Bangladesh; 3 Navrongo Health Research Centre, Ghana Health Service, Navrongo, Ghana

## Abstract

**Background:**

The 58th World Health Assembly and 56th WHO Regional Committee for Africa adopted resolutions urging Member States to ensure that health financing systems included a method for prepayment to foster financial risk sharing among the population and avoid catastrophic health-care expenditure. The Regional Committee asked countries to strengthen or develop comprehensive health financing policies. This paper presents the findings of a survey conducted among senior staff of selected Eritrean ministries and agencies to elicit views on some of the elements likely to be part of a national health financing policy.

**Methods:**

This is a descriptive study. A questionnaire was prepared and sent to 19 senior staff (Directors) in the Ministry of Health, Labour Department, Civil Service Administration, Eritrean Confederation of Workers, National Insurance Corporation of Eritrea and Ministry of Local Government. The respondents were selected by the Ministry of Health as key informants.

**Results:**

The key findings were as follows: the response rate was 84.2% (16/19); 37.5% (6/16) and 18.8% said that the vision of Eritrean National Health Financing Policy (NHFP) should include the phrases ‘equitable and accessible quality health services’ and ‘improve efficiency or reduce waste’ respectively; over 68% indicated that NHFP should include securing adequate funding, ensuring efficiency, ensuring equitable financial access, protection from financial catastrophe, and ensuring provider payment mechanisms create positive incentives to service providers; over 80% mentioned community participation, efficiency, transparency, country ownership, equity in access, and evidence-based decision making as core values of NHFP; over 62.5% confirmed that NHFP components should consist of stewardship (oversight), revenue collection, revenue pooling and risk management, resource allocation and purchasing of health services, health economics research, and development of human resources for health; over 68.8% indicated cost-sharing, taxation and social health insurance as preferred revenue collection mechanisms; and 68.75% indicated their preferred provider payment mechanism to be a global (lump sum) budget.

**Conclusion:**

This study succeeded in gathering the preliminary views of senior staff of selected Eritrean ministries and agencies regarding the likely elements of the NHFP, i.e. the vision, objectives, components, provider payment mechanisms, and health financing agency and its governance. In addition to stakeholder surveys, it would be helpful to inform the development of the NHFP with other pieces of evidence, including cost-effectiveness analysis of health services and interventions, financial feasibility analysis of financing options, a survey of the political and professional acceptability of financing options, national health accounts, and equity analyses.

## Background

Eritrea is situated in the Horn of Africa [1], and had an estimated population of 5.073 million people in 2009 [2]. The gross national income per capita was Int$643 in 2010 [3]. In 2007, 53% of the population was living below the national income poverty line of *Nakfa* 240 per capita per month [4].

The country has six health regions (Anseba, Debub, Southern Red Sea, Gash Barka, Maakel and Northern Red Sea) and 54 health districts. The health infrastructure comprises 369 health facilities, including 13 tertiary hospitals, 13 secondary hospitals and 343 primary level facilities (health centres, health stations, Maternal and Child Health units and Clinics, clinics and health posts) [5]. There are 292 licensed private pharmaceutical institutions, including 33 pharmacies, 31 drug shops and 228 rural drug vendors [6]. The health infrastructure is operated by 215 physicians, 2505 nursing and midwifery personnel, 16 dentistry personnel, 107 pharmaceutical personnel, and 88 environment and public health workers [7].

The total per capita expenditure on health in Eritrea was US$10 in 2008. About 44.9% of total health expenditure came from general government expenditure, the remaining 55.1% coming from private expenditure in the form of household out-of-pocket payments. The external resources for health made up 60.8% of total expenditure on health in 2008; i.e. channelled through public and private sectors [7]. Therefore, health funding came from government tax revenues, households’ out-of-pocket payments for health, and donor funding.

Over reliance on direct out-of-pocket payments for health care may expose households to the risk of financial catastrophe [8,9]. In order to mitigate that risk, the 58th World Health Assembly [10] and 56th WHO Regional Committee for Africa [11] adopted resolutions urging Member States to ensure that health financing systems included a method for prepayment to foster financial risk sharing among the population and avoid catastrophic health-care expenditure.

In 2006, Eritrean Ministry of Health developed its National Health Policy (NHP) whose vision is [12]:

“…the improvement of the health status of Eritrean people by creating an enabling environment for the provision of sustainable quality health care that is acceptable, affordable and accessible to all citizens” (p.15).

Three of the objectives of the NHP are related to health financing: (a) ensure that quality health services are available, accessible and affordable to all the people in the country; (b) introduce a health-financing scheme that protects the people from catastrophic expenditures and ensures sustainability of the system; and (c) implement the concept of hospital autonomy [12].

In view of global and regional developments, and the development of the 2006 NHP, the Ministry of Health realized that its first health financing policy, which was developed in 1996 and subsequently revised in 1998, needed to be revisited [13]. Whilst the revised policy adequately covers various aspects of cost sharing through the levying of user fees, it does not explore other revenue collection mechanisms, revenue pooling and risk management through prepayment, or the resource allocation and purchasing functions of health financing.

The Ministry of Health requested external technical support to start the process of developing a comprehensive National Health Financing Policy (NHFP) that explores the three functions of health financing: revenue collection, revenue pooling and risk management, and resource allocation and strategic purchasing. The preparatory support focused on the review of existing in-country literature on health systems with a focus on health financing, an analysis of health expenditure trends, and a rapid key informant survey to get preliminary views from relevant government ministries on the extended process of policy formulation.

This paper presents the findings of the preliminary key informant survey conducted among senior staff of selected Eritrean ministries and agencies to elicit views on some of the elements likely to be included in a national health financing policy.

## Methods

### Health financing framework

**Figure 1:**
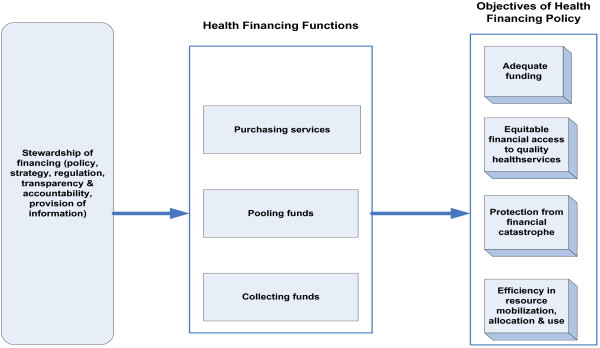
Health financing framework.

Figure 1 shows the framework of health financing which was adapted from Kutzin [14] and Thomson *et al.*[15]. That framework informed the design of the questionnaire used in the rapid key informant survey reported in this paper. The framework has four functions and four self-explanatory objectives.

According to WHO [16] stewardship involves ensuring strategic policy frameworks exist and are combined with effective oversight, coalition building, regulation, attention to system-design and accountability. This is an overarching function.

Revenue collection is the process through which the health system receives money, primarily from households, businesses, the ministry of finance and donors (in the form of grants and loans) [9]. Murray and Frank [16] describe eight mechanisms for mobilizing funds, namely: out-of-pocket payments, voluntary insurance rated by income, voluntary insurance rated by risk, compulsory insurance, general taxes, earmarked taxes, and donations from nongovernmental organizations and transfers from donor agencies [16].

Pooling has been defined by Gorret and Schieber as the accumulation and management of revenues so that members of the pool share collective health risks, and thus protect themselves from large, unpredictable health expenditures [17].

Purchasing refers to the mechanisms used to pay for health services from public, private-not-for-profit and private-for-profit providers [9]. The method of payment of service providers will likely affect both the quantity and the quality of health care provided to patients [18].

### Rapid key informant survey

A questionnaire entitled ‘*Eritrea national health financing policy questionnaire for government Ministries and Partners*’ was drawn up (see Additional file 1). The questionnaire was kept deliberately short to facilitate quick responses from busy senior government and partner agencies staff. It consisted of 11 questions meant to elicit respondent views on: the challenges and opportunities for health financing; key words that should be in the NHFP vision; objectives of the NHFP; core values that should guide NHFP; key components of the NHFP (e.g. stewardship, revenue collection, revenue pooling and risk management, resource allocation and purchasing of health services, development of human resources for health financing, health economics research, monitoring and evaluation); department in the Ministry of Health to be responsible for the implementation of the NHFP; whether the health financing agency should be a parastatal, private, or quasi-government agency; membership of the health financing agency board of directors; chair of the board meetings; and the secretary of the board.

The research team was advised by the Ministry of Health (MOH) leadership to focus the interview process on senior staff (Directors) in the MOH, Labour Department, Civil Service Administration, Eritrean Confederation of Workers (ENWC), National Insurance Corporation of Eritrea (NICE) and the Ministry of Local Government (MOLG). The reason for the restricted focus was the limited time available, and an understanding was reached with the MOH that broader consultations would take place at a later date. In this sense the people to be interviewed or consulted were purposely chosen by the MOH. A total of 19 key informants were interviewed or consulted. Out of those, 16 completed the questionnaire. Those who completed the questionnaire included 14 senior officials from the MOH; the Vice-Chairman of ENWC; and the General Manager of NICE.

Prior to completion of the questionnaires, the research team briefed the interviewees on the purpose of the survey and explained in detail the objectives and functions of health financing. This was done to ensure a basic understanding of the health financing framework.

#### Limitations of the survey

The survey was very limited in scope and did not include, for example, the MOF, health development partners in the country, and representatives of other stakeholder groups, such as civil society organizations, non-governmental organizations and the private sector.

## Results

About 84% (16/19) of the respondents completed the questionnaire. Table 1 shows the responses to the question: ‘*In your opinion, what are the key health financing related challenges facing Eritrea today?*’ The challenges mentioned by the respondents included: lack of a comprehensive NHFP; lack of a health planning office; shortage (insufficiency) of financial and human resources; increasing cost of medication; increased health awareness and demand for medical services; majority of population lives below the poverty line; lack of health insurance; lack of experience in operating a revolving fund; hospitals have no experience in retaining and managing user fees; poor referral mechanism; dearth of administrative and monitoring capacities; absence of sector-wide approaches for coordinating partner contributions; and inefficient use of available resources. The challenges most frequently mentioned included: lack of a comprehensive NHFP (5/16); and shortages (insufficiency) of financial resources (4/16) and human resources (3/16).

**Table 1:** The key health financing related challenges

**Challenges**	**Number of respondents**
Shortage (insufficiency) of financial resources	4
Shortage of skilled human resources	3
To develop a comprehensive HFP & start implementing it by phases	5
No insurance scheme	2
Majority of our population is below poverty line	2
No experience on revolving fund or retention of fund at service giving site	2
Facilities - hospitals not prepared to immediately take off the financing system. Poor management of resources is not to be denied.	1
Poor referral mechanism	1
Inefficient use of available funds	1
Unavailability of health planning office	1
Discouraging incentives	1
Absence of a SWAP for coordinating partner contributions	1
Dearth of administrative and monitoring procedures	2
Increasing cost of medication & awareness of medical facilities	1

Table 2 shows answers to the question: ‘*In your opinion, what are the existing local opportunities that Eritrea could leverage to strengthen the health financing system?*’ The respondents outlined a number of opportunities for strengthening the Eritrean health financing system, namely: high government commitment to good governance and accountability; high community participation in development activities; infrastructure (good hospitals with equipment and staff who can provide quality care); immediate need for reform of the hospital services and Government plans for making hospitals autonomous; cost-sharing programme; about 20% of the population are public servants with defined remuneration (payroll); willingness to pay for any good service; and absence of corruption in the public system. The high government commitment to good governance and accountability (4/16); high community participation in development activities (4/16); and willingness to pay for any good quality service (4/16) were the most frequently cited opportunities.

**Table 2:** Existing local opportunities to strengthen health financing system

**Opportunities**	**Number of respondents**
High government commitment	4
Community participation	4
Infrastructure (good hospitals with equipment & staff who can provide quality care)	2
Immediate need for reform of the hospital services	1
MOF should allow hospitals to use certain percentage of their revenue in hospital management	1
Introduction of cost sharing	1
Around 20% of the population is public servant with defined remuneration (payroll)	1
Willingness to pay is there for any good service expected	4
Free of corruption system	1
Tradition of paying tax	1

Table 3 summarizes responses to the question: ‘*What key words or phrases would you like to see in the vision of the Eritrean National Health Financing Policy?*’ The three most frequently mentioned phrases were: ‘equitable and accessible health services’ (6/16); ‘quality (plus continuous service improvement)’ (4/16); and ‘improve efficiency and reduce waste’ (3/16).

**Table 3:** Key words or phrases for inclusion in the vision of the national health financing policy

**Key words or phrases**	**Number of respondents**
High community participation	2
Quality (+ continuous service improvement)	4
Equitable and accessible health services	6
Social Insurance	1
Hospital Autonomy	2
Hospital Financing	1
How the Ministry should operate at some point in the future and how the population of Eritrea shall benefit from the health care system	1
Pro-poor/Increased government funding for the poor and disadvantaged	2
Cost containment	1
Increased affordability	2
Pricing policy	1
Improved efficiency & reduce waste	3
Increased government funding for priority diseases	1
Preventive measures	1
Transparency	1
Ensuring provision of a basic health package to all Eritreans and increasing coverage of quality health care for the poor	1
For sustainable development invest on health	1

Table 4 reports the responses to the query: ‘*Which of the following should be included as the objectives of the Eritrean NHFP?*’ A total of nine possible objectives were mentioned in the questionnaire, the following six being mentioned by more than 68% of the respondents:

(a). To secure a level of funding needed to achieve desired health goals and the objectives stated in the National Health Policy in a sustainable manner (15/16);

(b). To ensure efficiency in the allocation and use of health sector resources (14/16);

(c). To ensure equitable financial access to quality health services (13/16);

(d). To ensure that partner support for the implementation of the NHP is aligned, harmonized and coordinated (12/16);

(e). To ensure that people are protected from financial catastrophe and impoverishment as a result of using health services (11/16); and

(f). To ensure that the provider payment mechanisms chosen create positive incentives for providing quality preventive and curative services, responding to non-medical legitimate expectations of service users, and containing the cost of health care (11/16).

**Table 4:** Objectives of the national health financing policy

**NHFP objectives**	**Number of respondents**
To secure a level of funding needed to achieve desired health goals and objectives stated in the National Health Policy in a sustainable manner.	15
To ensure equitable financial access to quality health services	13
To ensure that people are protected from financial catastrophe and impoverishment as a result of using health services	11
To ensure efficiency in the allocation and use of health sector resources	14
To ensure that partner support for the implementation of the NHP is aligned, harmonized and coordinated	12
To ensure that provider payment mechanisms chosen create positive incentives for providing quality preventive and curative services, responding to non-medical legitimate expectations of service users, and containing the cost of health care	11
To empower providers to work towards privatization in phases	1
Ensure that the destitute part of the society has access to basic health services	1
May include exemptions for preventive and curative care for the segment of the population unable to pay	1

**Figure 2:**
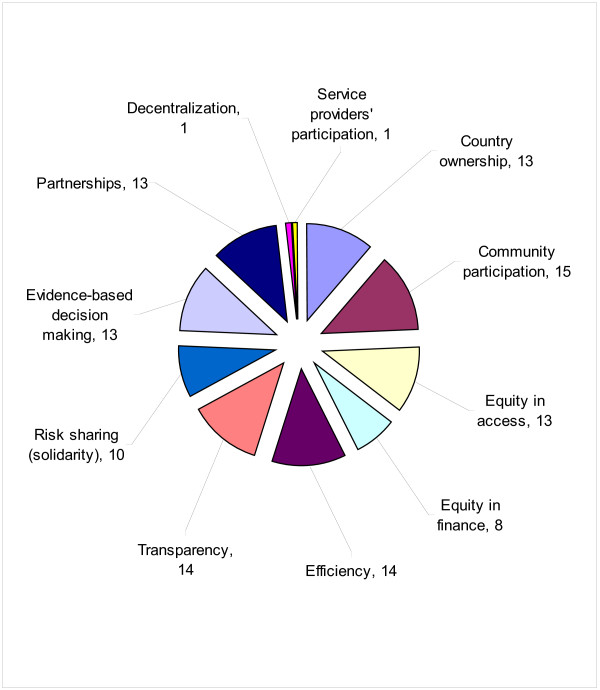
Number of respondents mentioning core values to guide national health financing policy.

Figure 2 encapsulates responses of sixteen MOH staff who responded to the question asking: ‘*Which of the following core values should guide the implementation of the NHFP?*’ A total of 11 possible core values or guiding principles were mentioned. Community participation, efficiency, transparency, country ownership, equity of access, evidence-based decision making and partnerships were mentioned by more than 80% of the respondents.

**Figure 3:**
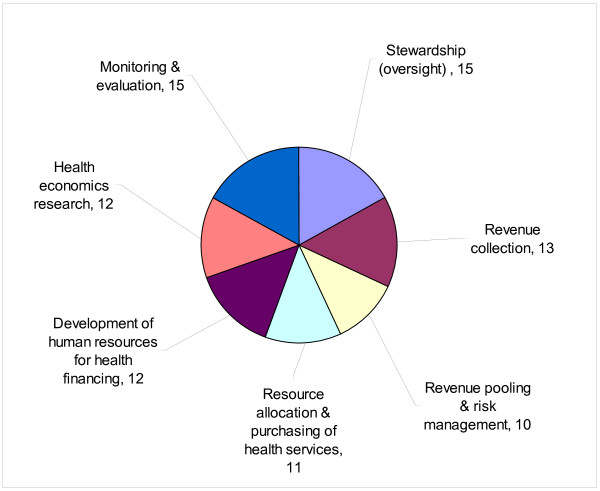
Number of respondents mentioning key components of national health financing policy.

Sixteen people responded to the question: ‘*In your opinion, which of the following should constitute the key components of the NHFP of Eritrea*?’ The responses are summarized in Figure 3. All seven components were mentioned by over 62% of the respondents. However, the three most frequently mentioned components were stewardship (oversight) for health financing (15/16), monitoring and evaluation (15/16) and revenue collection (13/16).

**Figure 4:**
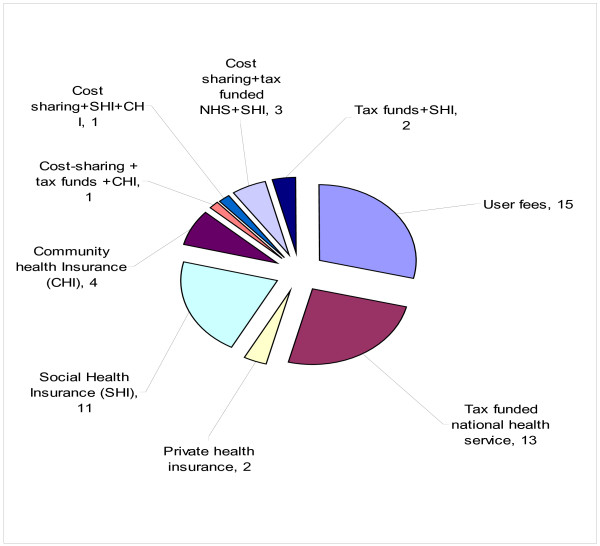
Number of respondents choosing revenue collection mechanisms.

Figure 4 provides a summary of answers of sixteen MOH staff who responded to the question: ‘*Taking into account the political, cultural and socioeconomic context of Eritrea, which of the following revenue collection mechanisms should Eritrea use?*’ The revenue collection mechanisms mentioned included: cost sharing through user fees; tax funded national health services; private health insurance; social health insurance; community health insurance and combinations of those mechanisms. However, the majority of the respondents thought that the preferred revenue collection mechanisms would be cost-sharing through user fees (15/16), tax funded national health services (13/16) and social health insurance (11/16).

**Figure 5:**
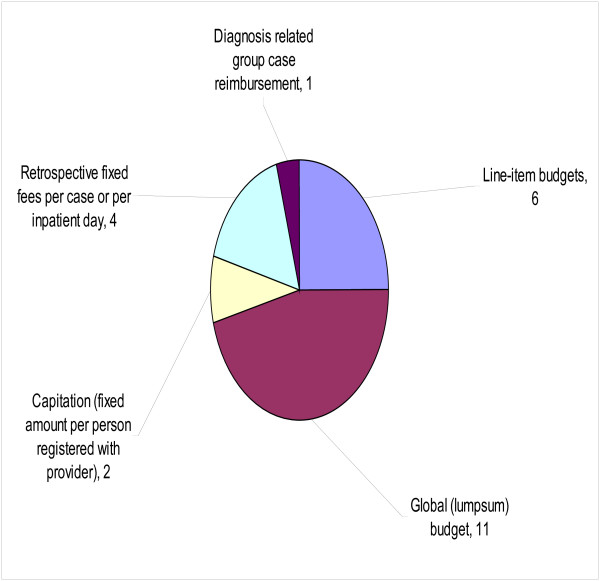
Number of respondents voting for provider payment mechanism.

Sixteen people responded to the question: ‘*Taking into account political, cultural and socioeconomic context of Eritrea, which of the following provider payment mechanisms should Eritrea use?*’ Figure 5 provides a summary of the responses to this question. The three provider payment mechanisms preferred by most people were: global (lump sum) budget (11/16); line-item budgets (6/16); and retrospective fixed fees per case or per inpatient day (4/16).

Twelve people responded to the question asking: ‘*In your opinion, which department of the Ministry of Health should be responsible for the implementation of the NHFP? Why?*’ The responses were as follows: Department of Administration and Finance (5/12); Department of Health Services (4/12); Health Systems Management Unit (HSMU) (1/12); Department of Clinical Services (1/12); and Departments of Health Services plus Department of Administration and Finance plus Planning Office (1/12). The respondents recognized that whichever department or unit takes the lead; it will have to collaborate with all other departments in the MOH. Of the eight people who gave reasons for their answers, five said that preferred Department/Unit was more related to the objectives of the NHFP; two indicated that the HSMU was responsible for NHFP development, implementation and monitoring; and one said that the Department of Clinical Services had better and closer contact with the service provider institutions.

Sixteen people responded to the question: ‘*In your opinion, what kind of agency should be established to manage complex links between risk management and both revenue pooling and purchasing of health services? Why?*’ Eighty one per cent of the respondents felt that a parastatal (government corporation) should be established to manage complex links between risk management, revenue pooling and purchasing of health services, while 3% felt that it should be handled by a quasi-government agency. None of the respondents said that a private agency would be appropriate. Eight of the respondents who gave a reason for their choice indicated: that Eritrea’s stage of development is appropriate for a parastatal (3/8); that parastatal or quasi-government agency status gives better autonomy as well as security (3/8); that a parastatal or quasi-government agency is appropriate because government primarily looks after the health of its people and will convince others to accept it (1/8); and that a parastatal or quasi-government agency is more appropriate for price and quality control.

All sixteen respondents addressed the question: ‘*Who should be members of the health financing agency board of directors?*’ As shown in Table 5, nineteen entities were named as potential members of the board of directors. Over 68% of the respondents indicated that the board of directors of the health financing agency should include: Minister of Health, Minister of Finance, Minister of National Development, Chairperson of the Eritrean Confederation of Workers, Chairperson of the Association of Employers, Director of the National Insurance Corporation of Eritrea, and a representative of the Medical Association of Eritrea. These responses clearly demonstrate a strong conviction among the MOH senior staff that the board of a health financing agency should have wide representation of all the stakeholders, e.g. relevant government ministries and parastatals, formal and informal sector workers (those with a union and those without), peasants, faith-based health service providers, private health-care providers, health development partners, health professional associations, and national women’s associations.

**Table 5:** Suggested members of the health financing agency board of directors

**Members of the board of directors**	**Number of respondents**
Minister of Health	16
Minister of Finance	15
Minister of National Development	13
Chairperson of the Eritrean Confederation of Workers	12
Representatives of formal sector workers who are not unionized, e.g. government employees	7
Chairperson of the Association of Employers	11
Director of National Insurance Corporation of Eritrea (NICE)	11
Representatives of the Faith-Based Organizations that provide health services	4
Representatives of the private health care providers	7
Representative(s) of the rural workers (peasants)	3
Representative of the informal sector workers	5
Vice-Chancellor of the University of Eritrea	1
Chairperson of the Sector-Wide Approach (SWAp) or the health development partner community	5
Representative of the Medical Association of Eritrea	11
Representative of the Nursing and Allied Health Workers Association of Eritrea	7
Representatives of Eritrean women association	1
ERIPA	1
Minister of Labour & Human Welfare	2
Minister of Local Government	1

Fifteen people responded to the question: ‘*Who should chair health financing agency board meetings?*’ Sixty-per cent (9/15) answered Ministry of Health; 27% (4/15) answered Ministry of Finance; 7% answered Minister of National Development; and 7% answered Minister of Labour and Human Welfare (pension head).

Twelve people responded to the question: ‘*Who should be the secretary of the health financing agency board?*’ Seven entities were named as possible sources of candidates. Thirty-three per cent answered Minister of Health, 25% (3/12) Minister of Finance, and 8% (1/12) either Minister of National Development, Chair of Sector-Wide approach (SWAp), Representative of the private health care providers, Chairperson or Director of National Insurance Corporation of Eritrea.

Finally, five people responded to the statement ‘*Kindly feel free to make other suggestions related to the development of the Eritrean NHFP.*’ Five suggestions were made. First, there is a need for statements regarding hospital autonomy and its implementation. Second, in-depth training of those people who are going to handle or implement the NHFP is essential. Third, as the private sector is not well developed at present the national health financing policy is expected to be developed with the focus mainly on public sector health care provision. Fourth, there is a need to ensure wider participation and consultation of relevant authorities and knowledgeable professionals. And last, since health financing is an integral part of social security, the health financing agency board ought to be chaired by the Social Security Division in the Ministry of Labour and Human Welfare (MoLHW).

## Discussion

### Key findings

There were seven key findings:

First, 37.5% (6/16) and 18.8% of the respondents said that the phrases ‘equitable and accessible quality health services’ and ‘improve efficiency or reduce waste’ should be included in the vision of Eritrean health financing policy (NHFP).

Second, over 68% of the respondents indicated that the objectives of the NHFP should include securing adequate funding, ensuring efficiency, ensuring equitable financial access, protection from financial catastrophe, and ensuring provider payment mechanisms create positive incentives to service providers.

Third, over 80% of the respondents posited that core values of NHFP should include community participation, efficiency, transparency, country ownership, equity in access, and evidence-based decision making.

Fourth, over 62.5% of the respondents stated that the NHFP components should include stewardship (oversight), revenue collection, revenue pooling and risk management, resource allocation and purchasing of health services, health economics research, and development of human resources for health.

Fifth, over 68.8% of the respondents suggested cost-sharing, taxation and social health insurance as preferred revenue collection mechanisms.

Sixth, 68.75% said that their preferred provider payment mechanism is a global (lumpsum) budget.

Seventh, 81% of the respondents opined that a parastatal (Government Corporation) should be established to manage complex links between risk management and both revenue pooling and purchasing of health services.

#### Vision

As already mentioned, the majority of respondents indicated that the NHFP vision statement should include the phrases ‘equitable and accessible quality health services’ and ‘improve efficiency or reduce waste’. Thus, a possible vision statement might read: ‘access of all population to needed essential package of quality promotion, prevention, treatment, and rehabilitation health services at an affordable cost and without waste’. This is close to the 58th World Health Assembly definition of universal health coverage (UHC) [10].

#### Objectives of the NHFP

The objectives cited as important by most respondents included: securing adequate funding; ensuring efficiency; ensuring equitable financial access; and protection from financial catastrophe. Securing adequate funding presumes the existence of a costed health sector strategic plan, estimates of available funding and any funding gap, and a resource mobilization strategy for bridging that gap. It is also important to note that ensuring efficiency entails the allocation and use of resources without waste, while equity in financing is dependent on household contributions to the funding of the health system being made according to ability to pay. Finally, protection from financial catastrophe requires payment long before health care is needed, and the pooling of resources to allow for the sharing of risk.

#### Core values

According to the majority of respondents the core values of the NHFP should include: community participation; efficiency; transparency; country ownership; equity in access; and evidence-based decision making.

Community participation requires recognizing communities as key partners in the planning, financing, implementation, monitoring and evaluation of health programmes and services [19]. Practically, the attainment of effective community participation in health development will require: the provision of an enabling policy/implementation framework for community participation; the strengthening of community capacities; the strengthening of the community-health services interface; the development and implementation of multi-sector policies and actions that facilitate community involvement in health development; and the promotion of healthy lifestyles in communities [20].

Efficiency requires minimizing the opportunity cost of attaining a given output or maximizing the output for a given opportunity cost [21]. In short, efficiency is about making the best use of available resources.

Transparency refers to openness in decision making and resource allocation. It may entail the strengthening of the parliamentary role in discussion and the adoption of national development strategies and/or budgets. In addition, it may involve: transparent results-oriented reporting and assessment frameworks; diagnostic reviews and use of the information derived from those reviews; and publishing of audit reports for public consumption [22,23].

Country ownership is about exercising leadership in the development and implementation of national development strategies through broad consultative processes [22]. With regard to financing this means ensuring that all health financing processes are led and owned by countries [11].

Equity in access requires that people in equal health need be treated equally regardless of their ability to pay or other irrelevant criteria (horizontal equity) and that people in unequal need be treated appropriately (vertical equity) [24].

Finally, evidence-based decision-making demands the use of relevant information when making health-care decisions at the level of policy, strategy, programme, system, planning, implementation, monitoring and evaluation [25].

#### NHFP components

According to majority of the respondents, the NHFP should include stewardship, revenue collection, revenue pooling and risk management, resource allocation and purchasing of health services, health economics research, and the development of human resources for health financing.

Stewardship in health financing involves oversight of all the other components of the NHFP. Therefore, the development of a health financing policy and strategy, legislation, management of health financing system and audit all have implications for stewardship.

The revenue collection mechanisms preferred by most our respondents included cost-sharing via taxation and social health insurance. Currently, the Eritrean health system is financed primarily through household out-of-pocket payments (OOP), tax revenues and donor funding. Therefore, it seems that the respondents envisage a shift from cost-sharing through OOP to prepayment through a mix of general taxation and compulsory contributions to health insurance. Although the contexts are very different, economically developing countries such as Eritrea may draw lessons from developed countries such as Austria, Belgium, Costa Rica, Germany and Luxemburg that have all made significant strides towards UHC via social health insurance [26].

According to Carrin and James [26], a number of factors facilitated those countries’ progress towards UHC via social health insurance: high per capita income that increased the capacity of businesses and citizens to prepay; a large formal sector that eased the capacity to contribute, and increased collection of contributions; urbanization and communication that facilitated the delivery of services; a skilled labour force to manage health financing schemes; the existence of solidarity within society that facilitated cross-subsidization from rich to poor, and from healthy to sick; government’s stewardship capacity to launch, guide and sustain a process of compulsory prepayment; and the availability of public and private health services of “acceptable” quality. Even though most of these attributes do not exist in the majority of African countries, the stories of Ghana [27,28] and Rwanda [28,29] offer some hope for countries contemplating the pursuit of UHC via a mix of general taxation and health insurance premium contributions.

Regarding *purchasing,* the majority of our respondents said that a global budget was their preferred provider payment mechanism. Culyer defines global budget as a method of reimbursing health service providers by establishing rates of payment in advance which are paid regardless of the costs in actual individual cases [21]. According to Altman and Cohen, global budgets are targeted to control total health care spending, i.e. the product of price and volume of health services [30]. Therefore, global budgets offer clear incentives for providers to control cost and operate efficiently. Of course, a potential problem is that providers who foresee a risk of exceeding their budget might resort to rationing by making patients to wait for long, which in turn may hamper access.

*Health economics research* entails reinforcing capacities for health financing evidence generation, dissemination and utilization in decision-making. Examples of pertinent health financing evidence include: costing; economic evaluation of services and interventions; economic viability analysis of health financing mechanisms; allocative and technical efficiency analyses and productivity trend analyses of health facilities; national and sub-national health accounts; and health financing equity analyses [31,32].

The *development of human resources for health financing* concerns the strengthening of financial management skills, including competencies in health accounting, auditing, actuarial science, health economics, budgeting, planning, monitoring and evaluation [11]. These competencies are a prerequisite in the design, development, implementation, monitoring and evaluation of a health financing system.

#### Agency

The respondents felt that a parastatal (Government Corporation) agency should be established to manage the complex links between risk management and both revenue pooling and purchasing of health services. Instead of setting up a new agency, The National Insurance Corporation of Eritrea (NICE) capacities could be reinforced to ensure the proper coordination of the three health financing functions. The governance (board of directors) arrangements of the agency should include representation from all relevant stakeholders mentioned by the majority of the respondents, e.g. relevant ministries, the Eritrean Confederation of Workers, the Association of Employers, the General-Manager of NICE, representatives of the medical and nursing associations of Eritrea, and representatives of agricultural workers and other non-unionized workers.

## Conclusion

The rapid key informant survey succeeded in gathering the preliminary views of senior staff of selected Eritrean ministries and agencies regarding elements likely to be an essential part of the NHFP, that is to say the vision, objectives, components, provider payment mechanisms, and health financing agency and its governance. The findings may be of help to the Ministry of Health in further refining the data collection instrument to be used in a more representative stakeholder survey.

In addition to an exhaustive stakeholder survey, there will be a need for a national health accounts study, a financial feasibility analysis of health financing options, an assessment of the political and professional acceptability of financing options, and equity analysis.

## Competing interests

The authors declare that they have no competing interests.

## Authors’ contributions

JMK, EZ and JA contributed to the study design, analysis and writing of various sections of the manuscript. All authors read and approved the final manuscript.

## Pre-publication history

The pre-publication history for this paper can be accessed here:

http://www.biomedcentral.com/1472-698X/12/16/prepub

## Supplementary Material

Additional file 1 **APPENDIX 1. **Questionnaire for Government Ministries and Partners. Click here for file
