# Supplementary material for: National health financing policy in Eritrea: a survey of preliminary considerations
Source: BMC Int Health Hum Rights. 2012 Aug 28;12:16. doi: 10.1186/1472-698X-12-16 (PMC3517356; doi:10.1186/1472-698X-12-16)
Supplement: Additional file 1 — APPENDIX 1. Questionnaire for Government Ministries and Partners. [file 1472-698X-12-16-S1.doc]

**APPENDIX 1: Questionnaire for Government Ministries and Partners**

**Introduction:** The Ministry of Health is in the process of revising the existing health financing policy to ensure that it comprehensive. In the revision process it is important to harvest the views of relevant government ministries and health development partners. Thus, we would greatly appreciate if you could help us to answer the questions below.

**National Health Financing Policy (NHFP) for Eritrea**

***Challenges***

1. In your opinion, what are the key health financing related challenges facing Eritrea today?
2. In your opinion, what are the existing opportunities (locally, regionally & globally) that Eritrea could leverage to strengthen her health financing system?

***NHFP***

1. What key words (or phrases) would you like to see in the vision of the Eritrean National Health Financing Policy?

***NHFP Objectives***

1. Which of the following should be included as the objectives of the Eritrean NHFP?
   1. To secure a level of funding needed to achieve desired health goals and objectives stated in the National Health Policy in a sustainable manner. [1=Yes; 2=N0]
   2. To ensure equitable financial access to quality health services. [1=Yes; 2=N0]
   3. To ensure that people are protected from financial catastrophe and impoverishment as a result of using health services. [1=Yes; 2=N0]
   4. To ensure efficiency in the allocation and use of health sector resources. [1=Yes; 2=N0]
   5. To ensure that partner support for the implementation of the National Health Policy is aligned, harmonized and coordinated. [1=Yes; 2=N0]
   6. To ensure that provider payment mechanisms chosen create positive incentives for providing quality preventive and curative services, responding to non-medical legitimate expectations of service users, and containing the cost of health care. [1=Yes; 2=N0]
   7. Others (specify):_______________________________________________________________________________________________________________________________________________________________________

***Core values or guiding principles***

1. Which of the following core values should guide the implementation of the NHFP?
   1. Country ownership [1=Yes; 2=N0]
   2. Community participation [1=Yes; 2=N0]
   3. Equity in access [1=Yes; 2=N0]
   4. Equity in finance [1=Yes; 2=N0]
   5. Efficiency [1=Yes; 2=N0]
   6. Transparency [1=Yes; 2=N0]
   7. Risk sharing (solidarity) [1=Yes; 2=N0]
   8. Evidence-based decision making [1=Yes; 2=N0]
   9. Partnerships [1=Yes; 2=N0]
   10. Others (specify):____________________________________________

***Policy components***

1. In your opinion, which of the following should constitute the key components of the NHFP of Eritrea?
   1. Stewardship (oversight) for health financing, e.g. development of pertinent legislation, strategic plan, guidelines, etc. [1=Yes; 2=N0]
   2. Revenue collection [1=Yes; 2=N0]
   3. Revenue pooling and risk management [1=Yes, 2=No]
   4. Resource allocation and purchasing of health services [1=Yes, 2=No]
   5. Development of human resources for health financing [1=Yes, 2=No]
   6. Health economics research and development [1=Yes, 2=No]
   7. Monitoring and Evaluation [1=Yes, 2=No]
   8. Other components (specify)

______________________________________________________________________________________________________________________________

1. Taking into account political, cultural and socioeconomic context of Eritrea, which of the following revenue collection mechanisms should Eritrea use?
   1. Cost-sharing through user fees [1=Yes, 2=No]
   2. Tax funded national health services [1=Yes, 2=No]
   3. Private health insurance [1=Yes, 2=No]
   4. Social health insurance [1=Yes, 2=No]
   5. Community health insurance [1=Yes, 2=No]
   6. Other (specify)______________
   7. Combinations (specify)_____________________________________.
2. Taking into account political, cultural and socioeconomic context of Eritrea, which of the following provider payment mechanisms should Eritrea use to reimburse hospitals (providers) for the cost of providing services?
   1. Line-item budgets [1=Yes, 2=No]
   2. Global (lumpsum) budgets [1=Yes, 2=No]
   3. Capitation (fixed amount per person registered with provider) [1=Yes, 2=No]
   4. Retrospective fixed fees per case or per inpatient day
   5. Diagnosis related group case reimbursement
   6. Others (specify):_____________________________________________

***NHFP Implementation***

1. In your opinion, which department in the Ministry of Health should be responsible for the implementation of the National Health Financing Policy. Why?
2. If Social Health Insurance were to be introduced, there may be need to set-up an autonomous health financing agency will be established to manage the complex links between risk management and both revenue collection and purchasing of health services.
   1. In your opinion, should the health financing agency be:
      1. Parastatal (government corporation),
      2. Private agency, or
      3. Quasi-government agency?

[Please provide reasons for your choice]

- 1. Who should be members of the health financing agency board of directors?
     1. Minister of Health [1=Yes, 2=No]
     2. Minister of Finance [1=Yes, 2=No]
     3. Minister of National Development [1=Yes, 2=No]
     4. Chairperson of the Eritrean Confederation of Workers [1=Yes, 2=No]
     5. Representatives of formal sector workers who are not unionized, e.g. government employees [1=Yes, 2=No]
     6. Chairperson of the Association of Employers [1=Yes, 2=No]
     7. Director of National Insurance Corporation [1=Yes, 2=No]
     8. Representative of the Faith-Based Organizations that provide health services [1=Yes, 2=No]
     9. Representatives of the Private Health Care Providers [1=Yes, 2=No]
     10. Representative(s) of the rural workers (peasants) [1=Yes, 2=No]
     11. Representative of the informal sector workers [1=Yes, 2=No]
     12. Vice-Chancellor of the University of Eritrea [1=Yes, 2=No]
     13. Chairperson of the Sector-Wide Approach (SWAp) or representative of the health development partner community [1=Yes, 2=No]
     14. Representative of the Medical Association of Eritrea [1=Yes, 2=No]
     15. Representative of the Nursing and Allied Health Workers Association of Eritrea [1=Yes, 2=No]
     16. Other (specify)_______________________________
  2. Who should chair the board meetings?
  3. Who should be the secretary of the board?

1. Kindly feel free to make other suggestions related to the development of the Eritrean National Health Financing Policy: ______________________________________________________________________________________________________________________________________________________________________________________________________THANK YOU FOR YOUR COOPERATION
